# Biofunctionalization of Natural Fiber-Reinforced Biocomposites for Biomedical Applications

**DOI:** 10.3390/biom10010148

**Published:** 2020-01-16

**Authors:** Tânia D. Tavares, Joana C. Antunes, Fernando Ferreira, Helena P. Felgueiras

**Affiliations:** Centre for Textile Science and Technology (2C2T), Department of Textile Engineering, University of Minho, Campus of Azurém, 4800-058 Guimarães, Portugal; taniatav13@hotmail.com (T.D.T.); joana.antunes@det.uminho.pt (J.C.A.); fnunes@det.uminho.pt (F.F.)

**Keywords:** natural fibers, biocomposites, surface modification, specialized biomolecules, immobilization methods

## Abstract

In the last ten years, environmental consciousness has increased worldwide, leading to the development of eco-friendly materials to replace synthetic ones. Natural fibers are extracted from renewable resources at low cost. Their combination with synthetic polymers as reinforcement materials has been an important step forward in that direction. The sustainability and excellent physical and biological (e.g., biocompatibility, antimicrobial activity) properties of these biocomposites have extended their application to the biomedical field. This paper offers a detailed overview of the extraction and separation processes applied to natural fibers and their posterior chemical and physical modifications for biocomposite fabrication. Because of the requirements for biomedical device production, specialized biomolecules are currently being incorporated onto these biocomposites. From antibiotics to peptides and plant extracts, to name a few, this review explores their impact on the final biocomposite product, in light of their individual or combined effect, and analyzes the most recurrent strategies for biomolecule immobilization.

## 1. Introduction

The use of eco-friendly materials has been increasing with time as a result of global environmental awareness. The development of recyclable and environmentally sustainable materials has become an attractive and important field of research. Natural fibers are among these materials and are gradually replacing synthetic fibers made from non-renewable petroleum-based resources [1,2].

Composites are formed of a strong load-carrying material (reinforcement) embedded within a “weaker” material (matrix). Because of the beneficial properties, abundance and low cost of natural fibers, these are considered a new generation of reinforcements for polymer matrices. By themselves, natural fibers are very unpredictable (with properties varying from batch-to-batch) and do not possess the mechanical resilience desirable for most applications; as such, combinations with polymer matrices have been proposed [3,4]. A biocomposite is considered a material that is composed of at least one natural resource. The natural fiber added value endows the biocomposites with a wide range of physical, mechanical and biological properties [5]. Manufacture of biocomposites can be accomplished by different processing techniques, including compression molding, injection molding, resin transfer molding, sheet molding, hand lay-up, filament winding, extrusion and pultrusion. These processes allow the natural fibers, which are presented in the form of loose fibers, nonwoven mats, aligned yarns and/or woven fabrics, to be placed in the desired direction to acquire specific mechanical properties in the final product [6]. There are other factors that must be considered as well to attain desirable properties, such as the type of natural fiber, the chemical compatibility between the fiber and matrix phases, the corresponding surface energies and the quality of the interface [7]. The interfacial bonding between both materials in a biocomposite are affected by the natural fiber’s hydrophilicity and polymer matrix hydrophobicity. Chemical and physical methods are required to treat the surface of the fiber to optimize this interaction [3].

The natural fibers’ abundance, availability and low-cost have made biocomposites very attractive for several industrial applications. However, in biomedicine, specific requirements must be met prior to their use. The most important is to be accepted by the human body without causing any adverse response, namely inflammation, allergies and/or early rejection associated with toxicity. Biocompatibility is, therefore, essential for the successful development of a biomedical device [8,9]. Even though biocomposites on their own have been reported in medical textiles [10], the addition of specialized biomolecules with particular properties, such as antimicrobial, anti-inflammatory, analgesic, sedative, anti-oxidative, UV-protection or chemical stability, to name a few, have demonstrated improved performance on specific biomedical applications. Biomolecules such as peptides, antibiotics, nanoparticles (NPs) or plant extracts functionalized onto biocomposites contribute significantly to their biocompatibility towards host cells, while improving other dormant material properties [11,12,13,14,15]. These combinations have been desirable for prospective applications in sutures, coatings for cell culture and drug delivery matrices, as well as for 3D scaffolds for ligaments, bone, cartilage, skin and vasculature engineering [10]. Still, even though they have demonstrated tremendous potential, research in this field is only now taking the first steps with the use of biocomposites for biomedicine, requiring further study and understanding. The present work explores this subject further by introducing some of the most recent (last ten years) biomolecule–biocomposite combinations and their final product properties. Fiber extraction, separation and chemical and physical processing prior to interfacial bonding with polymer matrices were also discussed. Finally, a detailed and critical analysis of the biomolecule’s inherent characteristics and the most recurrent methods employed for their immobilization onto natural fibers, fabrics and biocomposites was provided.

## 2. Natural Fibers

Natural fibers can be sourced from plants, minerals and animals [16]. The several physical and mechanical properties that characterize these fibers, such as low cost, low density, high specific strength and stiffness, processing flexibility, biodegradability and non-toxicity, allow an easy replacement of synthetic fibers [17]. Nowadays, plant-based fibers are very commonly used in many industrial sectors, such as textiles, automobiles, packaging, construction, sports equipment and medicine [3,18]. These are also known as ligno-cellulosic fibers, which can be extracted from inexpensive and available natural resources, and depending on the part of the plant from which they are sourced, can be classified into bast fibers (jute, flax, hemp, kenaf and ramie), seed fibers (cotton, milkweed, coir and kapok), leaf fibers (sisal, pineapple, agave, banana and abaca), grass fibers (sugarcane bagasse and bamboo), straw fibers (rice, corn and wheat) or wood fibers (softwood and hardwood) [2,16,18,19]. There are other natural fibers that are considered regenerated fibers, meaning that are produced from natural sources with human interference. Soybean is an example of this type, which undergoes chemical manipulation to be turned from a plant into a fiber [20]. Silk, wool, hair and feathers are examples of animal-based fibers composed mainly of proteins and are the second most important source of natural fibers [2,21]. However, compared to plant-based fibers they are stronger and more bioactive. Because of their high costs and lower accessibility, their use is restricted to biomedical applications [8,22]. In this field, natural fibers have attracted a research interest towards potential applications [23]. Medical textiles can be used from a simple gauze for wound dressings to sutures, reconstruction and repair of tissues and bones [24]. The materials for medical purposes require very specific characteristics, such as biodegradability, biocompatibility, functionability, bioresorbability, sterilizability, manufacturability, as well as mechanical properties [9]. Table 1 shows the mechanical properties of potential natural fibers for biomedical applications compared to human tissues.

In the last years, the use of natural fibers as reinforcement of composites has received considerable attention as substitutes of glass, ceramic and metal-based materials in various industries [1,17,18]. The application of these fibers has started in the automotive and aircraft sectors. However, nowadays they are being used in electrical and railway devices, as well as in civil engineering for structural and infrastructure applications such as roofs and bridges [16,19,26]. Biocomposites consist of a polymer matrix embedded with natural fibers; however, their binding is considered a challenge because of the numerous chemical structures of both the fibers and the polymers. Their performance depends on the properties of the individual components and their interfacial compatibility. Thus, it becomes necessary to modify the natural fibers resorting to specific treatments. Generally, the composition of the fiber structure is changed using reagent functional groups [1]. The reinforcement of a synthetic polymer with treated natural fibers introduces a positive effect on their mechanical and tribological performance. However, this performance depends of type, fraction or treatment of the fibers, type of polymer or manufacturing process [3,22]. Commonly, increasing the natural fiber amount in a polymer matrix leads to increased mechanical properties [8]. The matrix material is responsible for binding and protecting the natural fiber since, due to their fibrous nature, they cannot be used by themselves to sustain considerable loads [26].

### Fiber Separation and Extraction

Fiber separation and extraction are very important as they can affect the fibers’ quality, yield, chemical composition, structure, etc. [7,27]. Commonly, the separation of the plant-based fibers from the fiber crops is made by the retting process. This method consists in removing non-fibrous tissues attached to fibers through decomposition and degradation of hemicellulose and pectins, releasing individual fibers [2,28]. Table 2 compares five types of retting processes, namely dew, water, mechanical, enzymatic and chemical retting. Traditional methods, dew and water retting, rely on biological activity of microorganisms from the soil and are the most commonly used [29,30,31]; however, these have several disadvantages (Table 2). To overcome these limitations, improvements in fiber processing techniques are crucial to ensure consistently high-quality fibers and reduced environmental impact in terms of water waste and energy consumption [27,29]. Apparently, there is no single method that can give optimum results in all aspects. Enzymatic retting has been demonstrated to be the most promising solution due to its high enzyme specificity, better controllability, shorter duration and low environmental impact [32,33,34,35]. Nevertheless, the high cost of the process has not yet made it feasible at an industrial scale [7]. After the retting process, non-fibrous materials must be completely removed. For this, the fibers are extracted (breaking, milling, scutching or decortication), cleaned, refined and processed (spinning or weaving) to be used in a specific application [27].

Animal-based fibers come from diverse sources and, as such, the extraction occurs in different ways. The most used, silk, is obtained from silkworm cocoons that are composed of fibroin (fiber) and sericin (gum) proteins endowed with different biological and physicochemical properties [36,37]. Sericin is responsible for coating and protecting the fibroin, which needs to be extracted to release the fibers. Degumming is a process during which sericin is removed by thermo-chemical treatment of the cocoons, by boiling in a mild soap solution that dissolves the sericin gum binding the fibers and untangles them. Lastly, it is washed in cold water to remove the remaining sericin and other contaminations [8,38,39]. Wool fibers from sheep are, probably, the most widely used at an industrial scale and are mainly composed of keratin. Fiber extraction is accomplished manually by shearing and collecting “wool grease”, which has many impurities that must be washed and removed to extract clean wool [2,21]. Chicken feathers are also composed of keratin. The extraction of these fibers, composed of fiber (keratin) and quill, is initiated with a wash in water and ethanol to remove dirt and other particles present on the feather surface and dried under natural light. Then, barbs are mechanically separated from the quill, treated with NaOH, and further washed and dried [40,41,42].

## 3. Treatments of Natural Fibers for Successful Biocomposite Production

As mentioned earlier, it is important to modify the natural fiber surface to achieve a good interface bonding with the polymer matrix. Because of their low water and moisture absorption, and wettability [45], natural fibers require further chemical and surface treatments to optimize their performance as reinforcement agents.

### 3.1. Chemical Treatments in Plant-Based Fibers

Plant-based natural fibers are composed of cellulose, hemicellulose, lignin and wax [46]. Table 3 shows the percentage of chemical compounds in some of the most common natural fibers. Cellulose is the strongest and stiffest component of the fibers, endowing the fiber surface with several hydroxyl (-OH) groups and making them hydrophilic in nature. In addition, waxy substances cap the fiber reactive functional groups acting as an interference to interlock with the matrix that results in poor interfacial interaction with the hydrophobic polymer matrix. To turn the fibers less hydrophilic and, consequently, increase their mechanical and physical properties, modifications are necessary. Generally, the fiber structure composition is altered by chemical treatments using functional groups to react with the surface available hydroxyl groups. This can be accomplished through [3,45,46,47,48,49]:

Cellulose alkalization by removing the remaining fiber components (hemicellulose, lignin and wax) with sodium hydroxide (NaOH), cleaning the surface and increasing its roughness to improve adhesion to the polymer matrix;

Silanization treatment forming silane groups that act as a fiber-matrix coupling agent, creating a siloxane bridge between them. Silanol (Si-OH) groups react with -OH groups of the fibers and the matrix functional groups;

Acetylation by introducing an acetyl group on the fiber surface. Here, the -OH groups react with the acetyl groups decreasing their hydrophilic nature;

Peroxide treatment by generating free radicals that react with the -OH groups of both fiber and polymer. This treatment requires an alkaline pre-treatment;

Benzoylation treatment using benzoyl chloride to treat the fibers and decrease their hydrophilic nature by replacing of -OH groups with benzoyl groups. In this method, an alkaline pre-treatment is required;

Potassium permanganate treatment by forming highly reactive permanganate ions that react with the -OH groups, generating cellulose-manganate to initiate graft copolymerization;

Stearic acid treatment by inducing the interaction between reactive carboxyl groups of stearic acid with the fiber -OH groups, and thus improving water resistance properties;

Isocyanate treatment by acting as a coupling agent between the fiber and the matrix. Isocyanate functional groups react with the cellulose and lignin -OH groups, forming a chemical linkage by means of strong covalent bonds;

Maleated coupling treatment by means of maleic anhydride, which is used to modify the fiber surface and the polymeric matrix, ensuring high compatibility between them. Maleic anhydride is grafted onto the polymer, becoming available to react with the cellulose -OH groups by means of hydrogen or covalent bonds.

Many other chemical treatments can be used to treat fibers in order to reduce the number of hydroxyl groups and improve the fiber adhesion to the matrix, including acrylation, acrylonitrile grafting, triazine, zirconate, titanate, sodium chlorite, fungal and enzyme treatment. Chemical treatments comprehend a class of the most important approaches to improve natural fiber adhesion to a polymeric matrix, modifying their microstructure, improving tensile strength, wettability, surface morphology and increasing the number of available chemical groups [46].

### 3.2. Chemical Treatments in Animal Fibers

Animal-based fibers are mainly composed of structural proteins; hence, specific chemical modifications must be employed to these fibers, including coupling reactions (cyanuric chloride-activated, carbodiimide and glutaraldehyde coupling), amino acid modification (arginine masking, sulfation of tyrosine and azo-modified tyrosine) and grafting reactions (tyrosinase-catalyzed and poly(methacrylate) grafting). The primary structure of silk fibroin (SF), the protein from silkworm, contains a repetitive sequence of glycine-alanine-glycine-alanine-glycine-serine amino acids, which self-assemble into an anti-parallel β-sheet structure. The crosslinking between β-sheets along the protein is done by means of strong hydrogen bonds and Van der Waals interactions that endows silk with excellent mechanical properties [36,54]. SF is widely used in biomedical applications. However, it is essential to modify the SF surface chemistry to better control the interaction between silk and the living systems. SF possesses many reactive functional groups that facilitate crosslinking with other polymers, thus increasing its use as a reinforcing fiber [21]. Due to the presence of several reactive amino acids in SF, chemical modifications via coupling and grafting reactions and amino acid modifications can be applied. Wool and chicken feathers are mainly composed of keratin, a structural protein similar to SF. The chemical structure of keratin is predominantly an α-helix in chicken feathers [55] and a super coiled polypeptide chain with an α-helix and β-sheet in wool [56]. These structures are tightly packed via cross linkages, hydrogen bonds, Van der Waals and electrostatic interactions.

Chemical modifications play an important role in fiber functionalization, improving existing physicochemical properties or incorporating new ones. The fiber protein amino acid residue side chains may be conveniently conjugated with a variety of chemical groups [57]. These modification methods can be classified into coupling reactions, amino acid modification and grafting reactions. Coupling reactions are mainly used to immobilize peptides, molecules and polymers in fiber proteins. Copper-catalyzed azide-alkyne cycloaddition reactions, cyanuric chloride, carbodiimide and glutaraldehyde are very effective coupling agents [58,59]. The amino acid modifications are made through arginine masking, which is used to regulate the surface charge, sulfation/oxidation of tyrosine, which causes the hydrolysis of the fiber protein [58], and azo-modified tyrosine that can be used to install small molecules into fiber protein, resulting in hydrophobic and hydrophilic derivatives [60]. The grafting reactions include tyrosinase-catalyzed grafting and poly(methacrylate) grafting. Still, the chemical treatments discussed in Section 3.1. may also be applied to these protein fibers when used as composite reinforcements due to their several reactive functional groups [61,62].

### 3.3. Physical Surface Treatments

In addition to the mentioned chemical treatments, it is also very common to improve the fibers’ surface through physical surface treatments. Some of these approaches are used to functionalize the natural fibers’ surface and consist of the use of plasma, ultrasounds and UV-light. Plasma treatment is one of the most common surface modification methods. Cold plasma treatment is required to remove the surface impurities which, consequently, induces modifications in the surface properties, such as wettability, flame resistance, printability, etc., and increases surface roughness leading to better mechanical interlocking and interfacial adhesion between the fiber and polymer [47,63]. The hydrophilic/hydrophobic surface character can also be changed with the incorporation of free radicals capable of reacting with oxygen or other gases [48]. Plasma is a partially ionized gas that reacts with the fiber surface. Plasma is generated by applying an electrical field between two electrodes, which transmit energy, accelerating the gas electrons that collide with neutral gas molecules or atoms under atmospheric pressure or in a vacuum. In the case of a plasma vacuum, the gas is introduced at a low pressure in a vacuum chamber causing ionization by means of atom removal or bond rupture, giving rise to free radicals and crosslinking. However, this method requires an expensive closed system and is considered a batch process [64,65]. The treatment with atmospheric plasma is more attractive for industry, as it allows the samples to be treated in situ rather than restricted to a vacuum chamber. It is a continuous and uniform treatment, reliable and reproducible [66]. The atmospheric plasma technique can be divided into different types of discharge, such as corona-discharge, dielectric barrier discharge, glow discharge and atmospheric pressure plasma jet.

Corona treatment is a process based on low-frequency discharges applied in two opposing electrodes and grounded metal roll. These discharges induce ionization of the nearby atmosphere generating plasma. The fiber is placed in the gap between the electrodes and is bombarded with high-speed electrons, inducing surface oxidation and increasing the amount of high reactive free radicals [64,67]. It is a low-cost process with low energy consumption and exhibits several advantages compared with others plasma treatments [48]. The dielectric barrier discharge (DBD) technique is similar to the corona treatment. However, here, there is one or more dielectric barriers in the path between the electrodes, acting as an insulator. These accumulate the transported charge and distribute it over the entire electrode area. The gas between the electrodes is not ionized and only serves as a reservoir to absorb the energy dissipated. The main disadvantage of DBD is that it is not completely uniform and has a short duration [68,69]. The atmospheric pressure glow discharge (APGD) is a more stable, uniform and homogeneous surface treatment than DBD. This technique is generated in helium or argon by applying low voltages through parallel conductive electrodes at higher frequencies. The glow of the discharge refers to the characteristic luminescence resultant from excitation collisions followed by de-excitation [63,70]. In the atmospheric pressure plasma jet (APPJ) there are two tubular metal electrodes separated by a gap. Between the electrodes, a quartz cylindrical tube is inserted where helium (or other gases) flows. The plasma is launched into the surrounding air in the form of a plume or bullet, directly into the sample. This process can provide a local and very precise treatment [64]. APPJ is suitable for industrial and research applications, namely treatment of heat-sensitive materials, biological material sterilization and several biomedical devices [71].

Ultrasound treatment, while not as common as plasma treatment, is also effective in surface modifications. This method causes the cavitation effect, which is the formation, by ultrasonic irradiation, of small collapsing bubbles that generate powerful shock waves. The impact of the shock waves on the fiber surface leads to surface peeling, erosion and particle breakdown. Cavitation is responsible for the physical and chemical effects of ultrasound in solid/liquid and liquid/liquid systems and is more effective in heterogeneous systems than homogeneous systems. The effect of ultrasound treatment is related to its frequency; at low frequencies, violent cavitation is produced, and the effects are highly localized. On the other hand, with high frequency, the cavitation is less violent due to the shorter lifetime of the bubbles [49,72,73].

Ultraviolet treatment is based on UV-light, an electromagnetic radiation with a potential energy source capable of promoting photochemical reactions in the molecular structure of the fibers’ surface [74]. UV-treatment is a clean and cost-effective process that can be used in industrial applications [48]. In addition to the processes described earlier, there are other physical methods of surface modifications, such as ozone treatment, gamma-ray irradiation treatment, laser treatment and ion beam treatment [47].

## 4. Biomolecules and Their Immobilization Methods onto Biocomposites

Incorporation of biological cues onto the filament surface, through immobilization of bioactive ligands, peptides, NPs, enzymes, plant extracts or essential oils (EOs), has been used to obtain effective and specific biological functions of the composition. Immobilization of yeast invertase onto polyethylenimine (PEI)-coated cotton flannel for food modifying processes is one of the earliest cases of a biologically functional natural fiber by means of surface modification [75]. On the other hand, cotton and wool fabrics bearing covalently attached alkylated PEI exerted high bactericidal and antifungal activity [76] for wound dressing production, being a first example of the medical use of textiles functionalized with bioactive compounds [77]. Since then, biomolecules of all kinds have been immobilized on and within biocomposite materials for a variety of biomedical applications, including therapeutics, diagnostics, wound healing, tissue engineering, etc. A list highlighting the most recent (last ten years) formulations of biomolecule-modified biocomposites and respective “final product” properties is provided in Table 4. For the purpose of this review, inorganic NPs were considered biomolecules due to the biological and biomedical impact of their combination with selected biocomposites.

In the following sub-sections, a detailed analysis of these promising bioactive molecules applied in the production or modification of natural fiber-reinforced composites (Table 4) is provided together with a brief introduction about the approaches or methodologies required to attain such modified biocomposites.

### 4.1. Bioactive Biomolecules

#### 4.1.1. Antibiotics

The discovery of penicillin and streptomycin in 1929 and 1943, respectively, foreshadowed the age of antibiotics [117]. In fact, only two years later, the first definition for antibiotics was proposed: “chemical substance of microbial origin that possesses antibiotic powers” [118]. This definition only included those antibiotics produced by microorganisms but did not consider those of synthetic origin or produced by other biological products of non-microbial origin (but still endowed with antagonistic effects on the growth of microorganisms) [119]. As such, acceptable variations of this definition have been proposed over the years.

**Figure 1:**
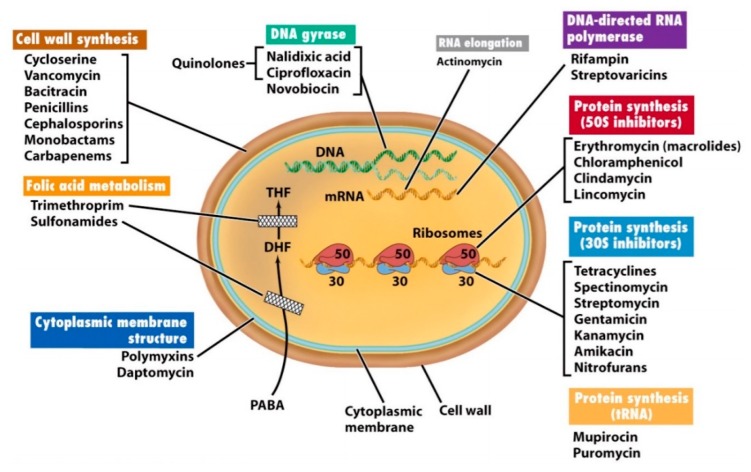
Antibiotic modes of action on bacteria (used with permission from [121]).

Currently, the antibiotics available in the marker are either produced by microbial fermentation or are synthetically prepared following the backbone structure of existing antibiotics. They target the physiology and biochemistry of bacteria (Figure 1) by affecting the membrane structure, the peptidoglycans or the cell wall biosynthesis; by interfering with protein synthesis via interaction with ribosomal subunits; by meddling with the DNA and RNA replication and transcription of nucleic acid synthesis and metabolism; and/or by interfering with metabolic pathways and, this way, inhibiting DNA synthesis. Ultimately, the effective action against these targets inhibits bacteria growth, compromises the cell integrity and, finally, leads to cell death [119,120]. The structural and metabolic differences between bacteria and mammalian cells enables antibiotics to induce selective toxicity against pathogens without harming the host cells [121].

For their efficiency and effectiveness, antibiotics represent a primary treatment method for infections and chronic diseases. However, the increasing and indiscriminate use of antibiotics has led to the development of tolerance and the emergence of antibiotic-resistant pathogens. In fact, this has become a serious global issue with devastating consequences for patient care [122]. The recognition of the correlation between antibiotic use and resistance development has catapulted research devoted to the discovery and design of new compounds effective against multi-drug-resistant pathogens and multi-organism biofilms [117,120,123]. In this context, many efforts have been made towards the design of new drugs, and the development of nanostructured platforms for the local and controlled delivery of antibiotics. One of the most common strategies consists in the immobilization of antibiotics at the surface of inorganic NPs or encapsulated within nano-sized shells [124]. Functionalization or modification of polymer-based composites has also been one of the most recurrent strategies in biomedicine [125].

With the concomitant rising interest in the use of renewable feedstocks, there has been great opportunities for the use of natural-origin materials in medical applications. Cellulose, for instance, is one of the most abundant polymers on Earth that can be harvested from natural fibers (Table 3). Butylparaben and triclosan antibiotics have been incorporated within the cationic β-cyclodextrin cellulose complexes cavities to improve the antibiotic’s solubility and, consequently, release kinetics. The antibiotic-loaded complexes were found to inhibited bacteria action by affecting the bacteria metabolism instead of damaging the cell membrane [126]. The incorporation of the ciprofloxacin hydrochloride antibiotic has also been attempted on a similar cellulose-based fibrous structure. β-cyclodextrin were covalently bonded to the cellulose fibers via citric acid, which prolonged the antibiotic release process and improved its antibacterial activity, particularly against *Escherichia coli* bacteria [127]. Research on the use of biocomposites as platforms for antibiotic delivery is fairly recent. Feather keratin/polyvinyl alcohol biocomposites have been produced by crosslink with dialdehyde starch for an improved compatibility. Dialdehyde starch was employed with the goal of decreasing the relative crystallinity and enthalpy of the composite, while increasing the water stability. Rhodamine B dye was used as a substitute of a model drug to explore the ability of this composite to sustain prolonged and stable drug release. Data confirmed this premise [128]. Research has continued on this subject and there are now woven cotton/polylactic acid composite systems loaded with amoxicillin [14], sericin (outer layer of silk fibers)/poly(vinyl alcohol) composites modified with tigecycline [78] and even keratin/hydrotalcite nanoparticle composites functionalized with diclofenac [79]. Acquired data shows the promising future of these new formulations and their ability to overcome the limitations of the use of free antibiotics, and their overall potential in biomedicine.

#### 4.1.2. Nanoparticles (NPs)

NPs are defined as solid colloidal particles of 1 to 100 nm in size and have been used in the biomedical field for a variety of purposes, including drug design and delivery, diagnostics and therapeutics. They can be engineered in the form of nanospheres, nanocapsules, liposomes, dendrimers and micelles from a variety of materials, including those from organic and inorganic origins [129,130]. The influence of NP parameters, such as size, shape, charge, colloidal stability, corrosion, stiffness and so forth, on interactions with molecules, living cells and animal models has been researched. However, interfacing inorganic NPs with biological settings have led to the most influential and outstanding discoveries [130,131,132]. For that reason, even though inorganic NPs are not considered biomolecules, their multiple biomedical applications and the various advantages offered when combined with biocomposites has led the authors to open an exception and include them in this section.

**Figure 2:**
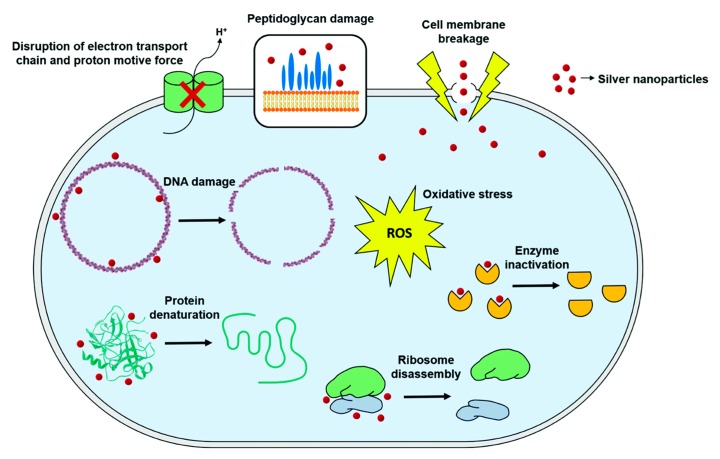
Overview of the antimicrobial action mechanisms of silver NPs (used with permission from [135]).

NPs are characterized by a large surface area-to-volume ratio. In the case of inorganic NPs, they can be subdivided into magnetic, metallic, bimetallic or alloy and metal oxide [133]. Much literature has focused on iron oxide NPs because of their superior chemical, biological and magnetic properties, including chemical stability, non-toxicity, biocompatibility, high saturation magnetization and high magnetic susceptibility. Maghemite (γ-Fe_2_O_3_) and magnetite (Fe_3_O_4_) are the most biocompatible oxidation states of iron. However, these forms tend to oxidize, requiring an additional coating made of other biocompatible materials, e.g., polymers [134]. Gold, silver and their respective compounds are the most widely employed metal NPs in biomedicine. Gold’s unique electronic and optical properties have resulted in important biosensor and bioimaging applications. Further, its easy functionalization with organic molecules allows for active or passive drug delivery systems to be engineered. Silver NPs are endowed with unique physicochemical properties that include high electrical and thermal conductivity, chemical stability, catalytic activity, enhanced optical properties and exceptional antibacterial performance. The antimicrobial activity of NPs, like silver, has been confirmed against a variety of microorganisms, including Gram-positive and Gram-negative bacteria and fungi. Because of their large surface area and reduced sizes, NPs can disrupt the cell wall and provoke membrane damage; penetrate intracellularly and cause protein denaturation, enzyme inactivation, DNA rupture or ribosome disassembly; and even induce oxidative stress (Figure 2) [135]. Silver NPs have contributed significantly to advances in medical textiles that include the production of wound dressings and protective coatings for medic devices [133]. In fact, the combination of these NPs with biocomposites is the most explored, with exceptional bactericidal properties being identified in cotton-, linen-, sugarcane bagasse-, silk- and jute-reinforced composites [13,81,82,83,84,85]. The bimetallic NPs comprehend those NPs that combine more than one metal or are produced from metallic alloys. Silver/copper NPs are a frequent example in this class. They have been used in the modification of cotton–polyester composites at different ratios and oxidation states with excellent antimicrobial properties against bacteria and fungi [86]. Metal oxide NPs are characterized by their unique physical and chemical properties and superior density. Size-related alterations in response to an increasing number of surface and interface atoms have been observed in NPs made of CuO, ZnO, SnO_2_, Al_2_O_3_, MgO, ZrO_2_, AgO, TiO_2_, CeO_2_, etc. Conjugation with biomaterial substrates has proven very effective in stabilizing these NPs and improving their performance. In fact, combinations with biocomposites have shown their harmless activity on human cells and improved antimicrobial action and UV-protection [97,98,99].

Derived from plant- and animal-based sources, organic NPs are highly biocompatible, nontoxic at various concentrations and often inexpensive. Most organic NPs are produced from natural-origin polymers, such as polysaccharides (e.g., chitosan, hyaluronic acid and cellulose) and proteins (e.g., albumin, elastin, collagen and silk). However, contrary to inorganic NPs, whose reproducibility is maintained with production, organic NPs have a significant batch-to-batch variability, displaying a range of physical and chemical properties that result from the poor control over the synthesis and fabrication processes. Because of that, very little reports have been published on the combination of these NPs with biocomposites [99,101,136].

#### 4.1.3. Enzymes: Laccase

Laccases, EC 1.10.3.2, p-diphenol:dioxygen oxidoreductase (60–100 kDa), are part of a larger group of enzymes termed multicopper enzymes that catalyze the oxidation of organic and inorganic substrates. Laccase is a glycosylated monomer or homodimer protein composed of carbohydrates like hexoamines, glucose, mannose, galactose, fucose and arabinose. To function, laccase depends on Cu atoms distributed among its three different binding sites.

Laccase was first described by Yoshida in 1883 and was then characterized as a metal containing oxidase by Bertrand in 1985, making it one of the oldest enzymes ever studied [137]. Laccases are widely distributed among plants, e.g., trees, cabbages, turnips, beets, apples, asparagus, potatoes, pears and other vegetables; insects of genera *Bombyx*, *Calliphora*, *Diploptera*, *Drosophilia*, *Lucilia*, *Manduca*, *Musca*, *Oryctes*, *Papilio*, *Phormia*, *Rhodnius*, *Sarcophaga*, *Schistocerca* and *Tenebrio*; and fungi, such as *Monocillium indicum*, *Cerena maxima*, *Coriolposis polyzona*, *Lentinus tigrinus*, *Pleurotus eryngii* and others from the *Trametes* species. Laccase activity has also been reported in few bacteria, including *Bacillus subtilis* [138]. Fungal laccase is perhaps the most widely researched, as its presence has been documented in virtually every fungus examined for it. Most fungi produce both intra- and extracellular enzymes, being the phenols, amines and benzoic acid, responsible for inducing the synthesis of laccase. Laccase can oxidize any substrate with characteristics similar to *p*-diphenol. Some fungal laccases are also capable of oxidizing monophenols and ascorbic acid. However, the primarily role of fungal laccase is to decompose lignin and/or to influence the polymerization of its oxidation by-products [137,139].

The activity of laccase-mediated systems is dependent on the redox potential of the enzyme and the stability and reactivity of the radical groups. Laccases are capable of catalyzing the mono-electronic oxidation of phenols and aromatic/aliphatic amines to reactive radicals and, simultaneously, reduce molecular oxygen to water in a redox reaction. Studies have shown that the phenolic sites of lignin macromolecules can be oxidized to phenoxyl radicals by laccase, and then undergo covalent coupling to initiate the polymerization of lignins. Laccase-oxidized phenols or non-oxidized amines can also be grafted to the radicalized lignins or lignocellulosic surfaces to produce engineered materials with novel functions [102,140,141]. As natural fibers, namely jute, are rich in lignin, the use of laccase to generate novel functions or induce stronger interfacial adhesion between non-polar resins in fiber-reinforced polymer biocomposites has been highly desirable [102,103,104].

#### 4.1.4. Peptides: RGD Motif

Peptides are versatile building blocks that adopt specific secondary structures, providing a unique platform for the design of self-assembling biomaterials with hierarchical 3D macromolecular architectures, nanoscale features and tunable physical properties. Various peptide motifs have been identified and used in biomedical applications [142]. However, the widely occurring arginine-glycine-aspartate amino acid sequence, also known as the RGD motif, is the most investigated. This simple tripeptide (75 kDa) endowed with cell adhesion properties (adhesion peptide) and located in the III_10_ module of the fibronectin protein is very complex and depends on flanking residues, the protein 3D structure and the individual features of the integrin-binding pockets [143]. For instance, by bonding with integrins, the RGD sequence allows fibronectin to assemble into fibrils and forming the primitive structure of the extracellular matrix. However, this motif is not restricted to fibronectin; indeed, it occurs within more than 100 proteins with either a cell adhesive activity or being functionally silent [143,144,145,146].

As pointed earlier, surface modification of biomaterials is of prime importance for biomedical applications, with biocompatibility being one of the major requirements (the material must be non-toxic to the relevant cells). Introduction of chemical stimuli, in the form of an RGD motif, along the biomaterial surface can facilitate its recognition and reception by the host cells. For that reason, functionalization by either inserting peptides coupled with binding agents or by embedding them into a polymeric matrix has been extensively researched and new bioactive biomaterials developed [147,148]. For instance, a composite of milkweed, polyethylene and polypropylene has been engineered and modified with the RGD peptide for bone replacement. The altered biocomposite was seen to promote MC3T3 osteoblast-like cells recruitment and, thus, to facilitate osteointegration [11]. Because of the particular functions and loads bone substitutes must endure, there is still much to be researched about the synergistic effect of natural fibers and the RGD motif. At this moment, most research on RGD-functionalized surfaces focus on metal-based biomaterials or polymer composites.

#### 4.1.5. Antimicrobial Peptides (AMPs)

**Figure 3:**
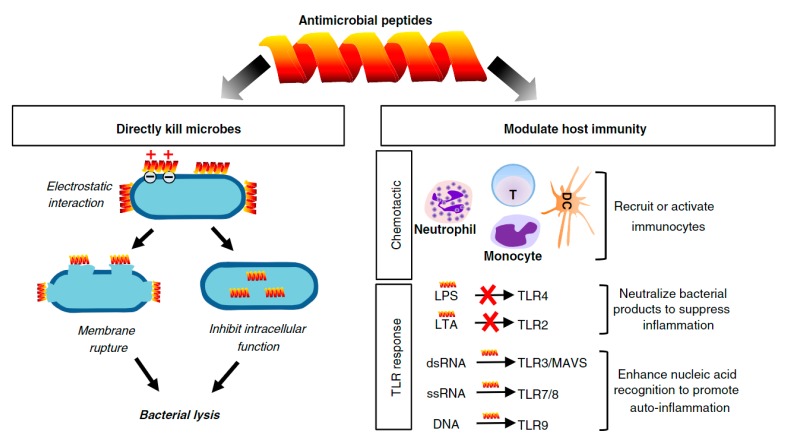
Biological functions of AMPs. AMPs bind to bacterial membranes through electrostatic interactions either to disrupt the membrane or to inhibit intracellular functions. Some AMPs also modulate host immunity by recruiting/activating immunocytes or by controlling the inflammatory response (used with permission from [157]).

AMPs are an integral part of the innate immune system, working as the first line of defense in a variety of organisms. They can be of natural or synthetic origin, are typically very short (5–100 amino acid residues), of low molecular weight (less than 10 kDa), positively charged (cationic with a net charge of +2 to +9) and amphiphilic. Most AMPs reported to date can be characterized as one of the following four types, based on their secondary structures: β-sheet, α-helix, extended and loop. Even though the β-sheet structure is the most common, it is only formed when the peptide comes in contact with a membrane [149,150,151,152,153]. In the case of natural origin AMPs, they can be isolated from both prokaryotes and eukaryotes. Most AMPs are produced by specific cells, at all times; however, there are those whose production is inducible. Still, they are quickly mobilized after microbial infection and act rapidly to neutralize a broad range of microbes (Figure 3) [149,150,153,154,155,156]. To date, hundreds of AMPs have been identified and their importance in the innate immune system explored.

Unlike antibiotics, which target specific bacteria cell functions (Figure 1), most AMPs target the microorganism’s lipopolysaccharide layer, which is exclusive to them. As eukaryotic cells are rich in cholesterol and possess a low anionic charge, they are out of the focus of many AMPs [151,154]. AMPs can be classified based on their target microorganism as antibacterial, which target bacterial cell membranes, compromising the lipid bilayer structure; antiviral, which neutralize viruses by integrating the viral envelope or the host cell membrane; antifungal, which kill by targeting either the cell wall or the intracellular components of fungi; and antiparasitic, which kill through direct interaction with the parasite cell membrane [149,157].

Functionalization of biomaterials with AMPs is a recent practice that is gaining much interest in the biomedical field. However, guaranteeing the antimicrobial performance of these peptides while immobilized remains a challenge, as it is dependent not only on the base substrate’s physical and chemical properties but also on the selected immobilization process. If blended with a polymer solution, for instance, the AMPs solubility can be compromised using organic solvents as they may deteriorate the biomolecules or induce aggregation, hindering their ability to penetrate or bind to the cell membrane. Cellulose acetate/poly(vinyl alcohol) composite films have been produced by solvent-casting followed by phase inversion for prospective applications in wound healing. The produced films were functionalized with LL37 by two methods, blending and surface binding via dopamine. Data reported a significant reduction of the LL37 antimicrobial action when immobilized by blending, proving the immobilization via binding agent more effective [158]. Physical binding methods, which include adsorption and layer-by-layer approaches, require the biomolecules dissolution prior to the physical adsorption by means of non-covalent or multidentate interactions [149]. Yet, this is not always feasible. A synthetic hybrid of cecropin and melittin has shown the tendency to form dimmers when in solution, augmenting its hemolytic activity and, thus, reducing its ability to penetrate the microbial membranes [159]. Still, when immobilized by covalent bonding on polyurethane-based substrates its action was significantly enhanced against Gram-positive bacteria [160]. Compared to physical binding methods, covalent immobilization offers many advantages, including minimizing AMPs leaching, providing long-term stability and lowering toxicity. Here, AMPs can be coupled to the surface via grafting, which requires covalent bonding of intact AMPs to the material surface, or via “surface initiated” methods, in which the synthesis of the AMPs is made through initiators of reactive groups covalently immobilized onto the biomaterials’ surface [149,155]. Because of their expensive and delicate nature, very little reports have been published on the functionalization of biocomposites with AMPs. One of the few works reports the modification of wool-based fibers with the Cecropin-B/[Ala5]-Tritrp7 hybrid AMP via the exhaustion method [12]. This modification improved the natural fibers’ antimicrobial action, both against Gram-positive and Gram-negative bacteria, and revealed the potential of these surfaces for biomedical uses.

#### 4.1.6. Plant Extracts

Plants are the most important source of natural drugs used in conventional medicine. Recent findings have demonstrated that near 72,000 (≈17%) of the 422,000 identified flowering species present a therapeutic potential. These values are continuously increasing since the bioactive molecules present in a plant species have also been identified in other plant species that are related with the former, thus increasing rapidly the diversity of plants that can be used in herbal medicine [161].

Plants produce proteins, lipids, carbohydrates and chlorophyll as the primary metabolic products after photosynthesis. These are easily found in nature, particularly in the seeds and vegetative tissues of tall plants. The secondary metabolites, however, are more difficult to identify and extract, being until recently discarded and their therapeutic potential ignored. These secondary biochemical pathways are capable of synthesizing raft chemicals in response to specific environmental stimuli, such as pathogen attacks [162]. Their roles comprehend the protection of the host by acting as antioxidant, free radical-scavenging, UV-light absorbing and antiproliferative agents, or by defending the plant against microorganisms such as bacteria, fungi and viruses [162,163]. The major classes of antimicrobial compounds extracted from plants are the phenolics, which antimicrobial action includes enzyme inhibition by the oxidized compounds (e.g., reaction with sulfhydryl groups) or through non-specific interactions with the proteins; the terpenoids (EOs), which give the plants their odors and are suggested to disrupt the membrane of bacteria, fungi, viruses and protozoa via lipophilic compounds; the alkaloids, which are heterocyclic nitrogen compounds capable of interfering with the DNA of pathogens by intercalating it; and the lectins and polypeptides, which are often cationic, thus allowing the formation of ion channels in the microbial membrane or inhibiting the adhesion of microbial proteins to host polysaccharide receptors by competing with those [164]. Mainstream medicine is increasingly receptive to the use of antimicrobial agents derived from plants as traditional antibiotics become ineffective [165]. As such, the incorporation of plant extracts onto polymeric-based substrates, natural fibers or even biocomposites are already widely investigated. Some of the most promising examples of the incorporation of plant extracts from different origins are highlighted in Table 4. Special consideration was given to natural fiber fabrics endowed with antimicrobial properties and to biocomposites with potential as regenerative medicine.

#### 4.1.7. Essential Oils (EOs)

Aromatic plants are very common in traditional medicine as antimicrobial agents. EOs are volatile, natural, complex compounds characterized by a strong odor that can be harvested from the essence of these aromatic plants [166]. In nature, EOs play antibacterial, antiviral and antifungal roles, as well as being insecticides, protecting plants against insects and herbivores or by reducing their appetite. They are also responsible for the attraction of specific insects that will then disperse pollens and seeds and promote the plant’s propagation [167,168]. EOs are produced by more than 17,500 species of plants from many angiosperm families, e.g., Lamiaceae, Rutaceae, Myrtaceae, Zingiberaceae and Asteraceae [169]. They are synthesized in the cytoplasm and plastids of plant cells, stored in complex secretory structures like glands or resin conduits, and only then presented as drops in the leaves, stems, flowers, fruits, bark and roots of the plants [170]. EOs are mainly composed of terpenes, terpenoids and phenylpropanoids at the levels of 20%–70% but may also contain fatty acids, oxides and sulfur derivatives [171]. They are generally obtained by steam- or hydro-distillation of plants. Their properties were first investigated by De la Croix in 1881 but since then many other researchers have analyzed the chemical composition and inherent properties of these volatile compounds [167,172]. In fact, the use of EOs in biomedicine has grown over the past four decades, being nowadays considered a potential alternative to antibiotics in the treatment of various infectious diseases. EOs are known for their antiseptic (bactericidal, viricidal and fungicidal), fragrance and medicinal properties, and have been employed in embalmment and preservation of foods and as antimicrobial, analgesic, sedative, anti-inflammatory, spasmolytic and local anesthetic remedies [173].

The EOs antimicrobial properties are frequently evaluated in light of their inhibitory or bacteriostatic effect against the replication of microbial cells or by their lethal or bactericidal activity. Their physiological role against microorganisms in not yet entirely understood; however, it is generally accepted that the spreading of EOs along the bacteria cell membrane enhances membrane permeability, which then leads to the subsequent loss of cell components. The acidification inside the cell blocks the production of ATP and leads to the coagulation of the cytoplasm and destruction of genetic materials (lipids, proteins, etc.) that, ultimately, lead to the cell death [167,173,174].

One of the major drawbacks associated with the use of EOs in biomedicine is their toxicity. It is well known that at high concentrations EOs may induce allergic reactions, thus being strictly regulated by the scientific committee on consumer products (SCCP). However, in the blood stream or in contact with eukaryotic cells the tolerance is even lower. As such alternatives for the controlled release of these volatile oils have been proposed. Recent studies have demonstrated that NPs functionalized with EOs have significant antimicrobial potential against multi-drug-resistant pathogens [175]. Other studies suggest the encapsulation of EOs onto chitosan to improve the antibacterial effect of the oils and their controlled release, without a toxic initial burst [176]. In this context, biocomposites of cotton modified with monochlorotriazinyl β-cyclodextrin, as an eco-friendly encapsulating/hosting compound, have been proposed for the formation of core-shaped hydrophobic cavities for individual loading of EOs [113]. The fibers contained in the peals of fruits like durian and coconuts have also been combined with synthetic polymers for the encapsulation of cinnamon [15] and oregano oils [116], respectively. Aside from enhancing the physical properties of the biocomposite, these EOs demonstrated improved antimicrobial action against Gram-positive and Gram-negative bacteria; this way attesting to the exceptional performance of EO-modified biocomposites.

### 4.2. Immobilization Methods

There are three major methods to immobilize biomolecules onto natural fibers: physical adsorption, physical entrapment and covalent attachment [77,177]. Physical adsorption includes: (1) van der Waals interactions, (2) electrostatic interactions, (3) hydrophobic effects and (4) affinity recognition [177,178,179]—all methods that imply self-organization (the molecules or ions adjust their own positions to reach a thermodynamic equilibrium) [180]. However, once adsorbed, the molecules may be further crosslinked to each other [177,178,179]. Van der Waals forces (including hydrogen bonding) are the most ubiquitous form of interaction between two material bodies, being caused by the electromagnetic fluctuations derived from the continuous movements of positive and negative charges within all types of atoms, molecules and bulk materials. They bring the bodies together. Through the use of stabilizing ligands or appropriate solvents, these interactions can be controlled to provide a useful tool with which to guide self-assembly [181]. Electrostatic forces hold ions together in an ionic compound [182]. They can be either attractive (between oppositely charged ions) or repulsive (between like-charged ions) and even directional, as in the case of structures with asymmetric surface-charge distributions or permanent electric polarization [181]. Electrostatic forces offer a type of bond that is low demanding in terms of the directionality and the distance between oppositely charged functional groups, having the least steric demand of all chemical bonds [183], in addition to the possibility of forming multi-center bonds [184]. Furthermore, the magnitude and length scale of these interactions can be regulated, namely by choosing the solvent (e.g., dielectric constant) and/or the concentration and chemical nature (e.g., size and valence) of the surrounding charged counterparts [181]. The use of these forces are a non-specific approach to immobilize biomolecules when the biomolecule has an isoelectric point higher or lower than seven and the surface a positive or negative charge [178]. Hydrophobic interactions involve separation of hydrophobic parts of amphiphilic objects from water molecules [180,181,185,186,187,188,189]. Hydrophobic interactions have been used to functionalize hydrophobic surfaces, using biomolecules like ligands attached to hydrophobic sequences. Surfaces with hydrophobic gradients have also been prepared [177]. But non-specific adsorption tend to provide little control in biomolecule orientation or activity, having low durability [178]. Finally, affinity interactions relate to the principle of complementary biomolecules interactions, by exploiting the selectivity of specific interactions (antibodies and antigens or haptens, lectins and free saccharidic chains or glycosylated macromolecules, nucleic acids and nucleic acid-binding proteins, hormones and their receptors, avidin and biotin, polyhistidine tag and metal ions). A marked advantage is their high selectivity, along with the possibility to control the orientation of immobilized biomolecules, high retention of the bioactive compound activity, mild reaction conditions and relative simplicity of the immobilization processes [178,181].

On the other hand, physical “entrapment” systems comprehend imprisonment of the bioactive compound within (1) microcapsules, (2) hydrogels, and (3) physical mixtures, such as matrix drug delivery systems [177]. Main advantages include simplicity, ability to use similar protocols for different biomolecules and simultaneous immobilization, stability and protection of the bioactive agent against degradation; while limitations comprise diffusion constraints (particularly with larger molecules) and the possibility of biomolecule leakage (if the entrapped molecule is small) [190,191]. The process of physical entrapment itself may also be harmful to the bioactive molecule [190].

**Figure 4:**
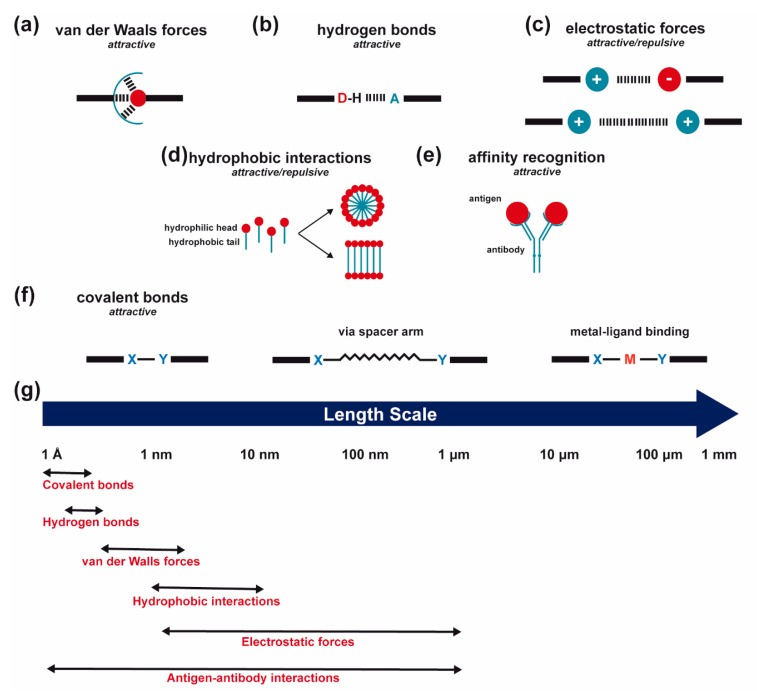
Forces involved in biomolecules immobilization onto natural fibers. (**a**) van der Waals forces; (**b**) hydrogen bonds between a H-bond donor and a H-bond acceptor; (**c**) electrostatic interactions between oppositely, or likely, charged species; (**d**) hydrophobic effects (here represented in the form of micelles or bilayers); (**e**) example of affinity recognitions, such as an antigen–antibody interaction; (**f**) covalent bond between donors X and Y without a spacer arm (left), via a spacer arm (middle) and metal–ligand binding between a soluble metal acceptor center (M) and organic ligand donors (X and Y) (right); and (**g**) length scales of the forces involved, taking into account that hydrophobic interactions occur upon contact, and that antigens are bound to antibodies through electrostatic interactions, hydrogen bonds, van der Waals forces and hydrophobic interactions [180,181,185,186,187,188,189,199,200].

Finally, covalent attachment comprises short-range intermolecular attractive forces at the molecular scale. Two electrons are shared by two atoms [181,182]. Covalent attachment may occur within a polymeric chain (water-soluble polymer conjugates), onto a solid surface or within hydrogels [177]. Chemical coupling reactions should achieve very high yields under mild conditions with few side reactions and little denaturation of the bioactive compounds [190]. Numerous covalent bonding chemistries exist. Regardless, a main advantage of a covalent bond is that the molecule is tethered at a site on its surface rather than in contact over a significant part of its surface as in the case of physical adsorption. The molecule is therefore generally more remote from the binding surface. Notwithstanding, covalent binding may excessively constrain the biomolecule or at least increase the probability of involving the bioactive site in the interaction with the surface. The proximity of the surface may also hinder the interaction between the bound molecule and other molecules in the solution [192]. For this reason, the inclusion of a spacer group (also called the linker, arm or tether) is often recommended to allow the tethered molecule to be located further from the tethering surface [177,192]. One of the most popular tethers is a poly (ethylene glycol) (PEG) molecule that can be derivatized with different reactive end groups [177,193]. Such spacers can provide greater steric freedom, and thus greater specific activity for the immobilized biomolecule. The spacer arm may also be either hydrolytically or enzymatically degradable, and therefore will release the immobilized biomolecule as it degrades [177]. However, the use of a linker does not always implies higher biomolecule activity, as the linker may adopt a conformation that interferes with the function of the compound [192]. Coatings with PEG, PEG derivatives like PEG-containing surfactants, other hydrogels, saccharides, proteins, choline headgroups and hydrogen bond receptors have also been useful to confer new functionalities to a surface, stabilize and protect the load and provide stealth effect at the host environment [178,194]. Of particular interest is the metal–ligand binding between a soluble metal acceptor center and organic ligand donors: Attractive coordination of covalent bonds that give rise to infinite metallo–organic architectures [195]. Both the metal and the ligand are typically chemically modified during bond activation, which depends on the nature of the metal and ligand structures. A metal–nitrogen bond is the most well-studied cooperation interaction, although metal–oxygen, metal–sulfur and metal–carbon also occur frequently [196]. Indeed, recently, a wide variety of metal−ligand bonds have been formed and used to functionalize metal NPs, beyond the conventional metal–thiolate (M-S) linkages. NP-mediated intraparticle charge delocalization is a unique advantage. In addition, chemical events that occur at a specific site on the NPs surface may be propagated and even amplified to all NPs, resulting in a clear variation of the NPs spectroscopic and electrochemical properties [197]. Metal-centered compounds with endless complex structures and shapes enable new chemistries, like novel mechanisms of action not accessible by organic small molecules, towards the discovery of new drugs. The metal and/or ligands can interact with nucleic acids or amino acid residues, inhibiting the function of a targeted biomolecule. Consequently, metal–ligand interactions are being increasingly studied for therapeutic applications [185,198]. A variety of physical properties (redox, optical and magnetic) are also presented by the metallic donors and allow suitable spatial and electronic arrangement for mild and selective bond activation processes, resembling highly selective bond activation reactions that occur in enzymes under mild conditions [185,196]. Figure 4 represents each of the latest referred intermolecular forces.

But irrespective of the method used, the same biomolecule may be immobilized by many different methods, plus more than one biomolecule may be immobilized to the same support. Major immobilization method trends comprise the exhaustion method, dip–pad–dry–cure method, covalent chemistry and in situ inorganic NP synthesis through the hydrothermal sol-gel method. Of interest is a successful biomolecule immobilization in a sufficient amount, along with retention of an acceptable level of bioactivity over an appropriate time period [177]. Table 5 summarizes recent examples of bioactive molecule immobilization strategies onto clean and/or pre-treated natural fibers.

## 5. Conclusions and Future Perspectives

Biocomposite materials are a relatively recent addition to the composites class, with desirable properties for biomedical applications. Along this review, the advances on this front were highlighted, and the improvements made by the introduction of attractive biomolecules and respective immobilization processes and the selection of specific fiber and/or surface treatments were analyzed in detail. It is now clear that the success of a biocomposite relies greatly on the compatibility of the individual materials and the interactions formed. Here, the pre-treatment of the natural fibers and the surface modifications are essential. Immobilization of biomolecules onto these biocomposites represents a step forward to their use in specific biomedical applications. Addition of drugs, NPs, peptides or even plant extracts were found to improve the biocompatibility and antimicrobial resistance of the biocomposites, qualities that are fundamental to a successful implantation. Still, challenges remain and should be properly addressed in future works. One of the major challenges lies with the understanding of the material’s individual properties and the proper selection of the processing tools. It is well known that the manufacturing process, and all the inherent stages of production, have an important influence on the final product. Because of their biological origin, the extraction of the natural fibers and their consequent properties are more difficult to predict. Hence, variations between batches are encountered, which may also explain why biocomposites developed in the laboratory have limited success during clinical trials.

Production of biocomposites for biomedical uses relies on a different set of rules than other composites for other applications. They need to be tailored and optimized to fit the desired local and global arrangement of the reinforcement phase so that the implantable biocomposite can become structurally compatible with the host tissues. Efforts should be made to harness the potential of textile biocomposites for the design of implants with improved performance. The modification of materials with a biomolecule of interest has been very important to reach this goal, particularly for external-use medical textiles. Yet, the long-term durability and reliability of internal-use biomaterials made from these biocomposites require further research efforts. In view of their clear potential, which is intimately related to their flexibility in introducing surface modifications via biomolecules, it is expected that biocomposite materials will find increasing uses in biomedicine.

## Figures and Tables

**Table 1:** Mechanical properties of natural fibers and human tissues comparatively (adapted from [1,2,8,16,19,25]).

	Tensile Strength (MPa)	Elongation at Break (%)	Young’s Modulus (GPa)
**Natural fibers**			
Jute (*Corchorus capsularis*)	393.0–773.0	1.5–1.8	13.0–26.5
Flax (*Linum usitatissimum* L.)	345.0–1100.0	1.3–10.0	27.6
Hemp (*Cannabis sativa*)	550.0–900.0	1.6	30.0–70.0
Kenaf (*Hibiscus cannabinus*)	295.0–1191.0	3.5	53.0
Ramie (*Boehmeria nivea*)	348.0–938.0	1.2–8.0	44.0–128.0
Cotton (*Gossypium* sp.)	264.0–800.0	7.0–8.0	5.5–12.6
Milkweed (*Calotropis gigantea*)	381.0	2.1	8.2
Coir (*Cocos nucífera*)	131.0–175.0	15.0–25.0	4.0–6.0
Kapok (*Ceiba pentandra*)	90.0–95.0	1.8–4.2	4.0
Sisal (*Agave sisalana*)	500.0–800.0	2.0–25.0	9.4–22.0
Pineapple (*Ananas comosus*)	170.0–1627.0	2.4	60.0–82.0
Agave (*Agave americana* L.)	430.0–580.0	3.0–4.7	13.2
Banana (*Musa sepientum*)	529.0–914.0	3.0	27.0–32.0
Sugarcane bagasse (*Saccharum officinarum*)	20.0–290.0	1.1	17.0
Bamboo (Bambusoideae)	140.0–230.0	--	11.0–17.0
Rice (*Oryza sativa*)	450.0	--	1.2
Corn (*Zea mays*)	160.0–175.0	--	4.5–5.1
Wheat (*Triticum* sp.)	275.0	--	4.5–6.5
Softwood (different species)	1050.0	--	40.0
Hardwood (different species)	1000.0	--	38.0
Silk (*Bombyx mori*)	650.0–750.0	18.0–20.0	16.0
Wool (*Ovis aries*)	120.0–174.0	25.0–35.0	2.3–3.4
**Human tissues**			
Hard tissue (e.g., tooth, bone)	130.0–160.0	1.0–3.0	17.0–20.0
Skin	7.0–6.0	78.0	--
Tendon	53.0–150.0	9.4–12.0	1.5
Elastic cartilage	3.0	30.0	--
Heart valves	0.5–2.6	10.0–15.3	--
Aorta	0.1–1.1	77.0–81.0	--

**Table 2:** Properties and limitations of the five types of retting processes.

Retting Type	Description	Advantages	Disadvantages	Duration of Retting	References
Dew Retting	Plant stems are cut and distributed in the field exposed to the action of pectinolytic microorganisms that disrupt pectins surrounding the fiber.	Low cost and sustainable process.	Influenced by uncontrollable weather conditions and soil-contaminated fibers; reduces fiber strength, consistency and quality.	2–10 weeks	[2,7,31,43]
Water Retting	Plant stems are submerged in water (river, ponds or tanks) where anaerobic bacteria develop and break down the pectins.	Produce uniform and high-quality fibers.	Large consumption and contamination of water (superior environment impact); extensive stench of fermentation gases and high labor costs.	7–14 days	[2,7,29,30]
Mechanical Retting	The fibers are separated by mechanical means, such as a decorticator or hammermill.	Simple process that produces huge quantities of fiber in a short retting time.	High cost and lower fiber quality.	2–3 days	[2,27,43]
Enzymatic Retting	Fiber separation is made using pectin-degrading enzymes (pectinases) in a bioreactor.	The process is done under controlled conditions, is fast and clean; produces high-quality and consistent fibers.	High cost	8–24 h	[7,27,28,33,34,35]
Chemical Retting	Pectins are removed from the plant by dissolution in water tanks filled with chemical solutions.	The process is unaffected by weather conditions and can produce consistent and high-quality fibers in short times.	High processing cost and consumption of water, chemicals and energy (superior environment impact).	75 min–1 h	[2,7,44]

**Table 3:** Chemical composition of some of the most common natural fibers (adapted from [1,2,27,45,50,51,52,53].

Fiber	Cellulose (wt %)	Hemicellulose (wt %)	Lignin (wt %)	Wax (wt %)
**Bast fibers**				
Jute	61.0–71.5	13.6–20.4	12.0–13.0	0.5
Flax	71.0	18.6–20.6	2.2	1.7
Hemp	70.2–74.4	17.9–22.4	3.7–5.7	0.8
Kenaf	45.0–57.0	21.5	15.0–19.0	--
Ramie	68.6–76.2	13.1–16.7	0.6–0.7	0.3
**Seed fibers**				
Cotton	82.7–91.0	5.7	--	0.6
Milkweed	55.0	24.0	18.0	1.0–2.0
Coir	32.0–43.0	0.2–0.3	40.0–45.0	--
Kapok	13.0–35.0	23.0–32.0	13.0–21.0	--
**Leaf fibers**				
Sisal	67.0–78.0	10.0–14.2	8.0–11.0	2
Pineapple	70.0–82.0	--	5.0–12.0	--
Agave	68.4	4.9	4.9	0.3
Banana	63.0–64.0	6.0–.0	5.0	--
Abaca	56.0–63.0	20.0–25.0	7.0–12.4	3
**Grass fibers**				
Bagasse	55.2	16.8	25.3	--
Bamboo	26.0–43.0	30.0	21.0–31.0	--
**Straw fibers**				
Rice	41.0–57.0	33.0	8.0–19.0	8.0–38.0
Corn	38.0–40.0	28.0	7.0–21.0	--
Wheat	38.0–45.0	15.0–31.0	12.0–20.0	--
**Wood fibers**				
Softwood	40.0–45.0	7.0–14.0	26.0–36.0	--
Hardwood	38.0–50.0	19.0–26.0	20.0–30.0	--

**Table 4:** Application of biomolecules in the production of natural fiber-based composites for potential biomedical applications and respective properties. Most of the selected combinations have already been established for biomedical uses. However, there are a few that, even though the publications do not state those as potential applications, the authors feel that the combinations or the principles described may be of interest for biomedical uses and as such were included. This table compiles examples of natural fiber-reinforced composites modified with multiple biomolecules reported in the last 10 years.

Category	Specific Biomolecule		Natural Fiber-Reinforced Composites	Biofunctionalized Fibers/Fabric/Composite Production and Properties	References
	Name	Characteristics			
**Drugs/Antibiotics**	Amoxicillin	Penicillin-type antibiotic that works by stopping the growth of bacteria. Used to treat several bacterial infections like, middle ear infection, strep throat, pneumonia, skin and urinary infections, etc.	Woven cotton fabric/polylactic acid composite	Drug-loading capacity increased with decreasing fabric porosity. Degradation of the fabric composites influenced drug release rate. Water absorption decreased with increasing PLA concentrations. The mechanical properties of the composites were consistent with the fabric’s density and weight.	[14]
	Tigecycline	FDA approved glycylcycline antibiotic used in the treatment of skin tissue infections.	Sericin (outer layer of silk fibers)/poly(vinyl alcohol) composite	Composite fibers showed a smooth and uniform morphology with suitable porosity, mechanical stability and water vapor transmission rate. They also revealed antibacterial activity against *Escherichia coli* and *Bacillus subtilis*. In vivo testing showed this composite to accelerate wound healing.	[78]
	Diclofenac	Nonsteroidal anti-inflammatory drug used to treat pain and inflammation associated with arthritis.	Keratin/hydrotalcite NPs composite	Keratin extracted from wool and filled with hydrotalcite NPs intercalated with anionic diclofenac gave rise to a new composite. These showed a less pronounced swelling, porosity and degradation and a greater thermal stability compared to pure keratin films. Diclofenac release profile was more stable on the modified composites, which were also able to support fibroblast-like cells adhesion.	[79]
	Dimethyl phthalate	Colorless liquid soluble in organic solvents, commonly used as an insect repellent and ectoparasiticide.	Sugarcane bagasse/starch granules composite	Cellulose nanofibers derived from waste sugarcane bagasse were mixed with starch granules to produce a low porosity biocomposite with enhanced water uptake. The initial dimethyl phthalate release burst was reduced, gaining a superior controlled release efficiency overtime.	[80]
**Nanoparticles (NPs)**	Silver (Ag)	Inorganic particles endowed with superior antimicrobial activity. Their mechanism of action is not yet completely understood but it is clear it is significantly affected by the particles’ nanoscale dimensions.	TEMPO (2,2,6,6-tetramethylpiperidine-1-oxyl radical) selectively oxidized jute fiber	AgNPs, averaging 50.0 ± 2.0 nm, were formed in situ and deposited on the surface of jute cellulose fibers by microwave heating. The versatile jute-AgNPs nanocomposites demonstrated superior thermal stability and high crystallinity.	[81]
			Silk fibers/polyhexamethylene biguanide (PHMB) fabric	Regenerated silk fibers were fabricated through the dry–wet spinning process and modified via master batch or dipping process with different concentrations of PHMB and AgNPs. The bactericidal efficiency of the master batch treated fabrics was dependent on the concentration of the antibacterial agent as well as particle size. In the dipping process, a compromise was made between the good inhibition effect and the least amount of color change on the bio-fibers.	[82]
			Sugarcane bagasse/acrylamide/glycidyl methacrylate composites	Sugarcane bagasse was successfully grafted with acrylamide and glycidyl methacrylate and further modified in a colloidal suspension of AgNPs, gaining superior antimicrobial action against *Escherichia coli*, *Staphylococcus aureus*, *Aspergillus flavus* and *Candida albicans*.	[83]
			Linen (from flax family)/chitosan composite	Linen fabrics coated with chitosan and modified with AgNPs via in situ synthesis with tamarind seed coat extract showed efficient multifunctional properties, with bacterial reduction of 100%, UPF rating of 50+ and antioxidant activity of 97%. Except for flame retardancy, all properties were retained to a satisfactory level even after 50 washing cycles.	[84]
			Cotton/carboxymethyl chitosan/L-cysteine composite	Cotton fabric grafted with carboxymethyl chitosan and immobilized with AgNPs, via amidation reaction with the L-cysteine groups available at the fabric surface, demonstrated enhanced antibacterial functions, sustained even after 180 cycles of washing. Cytotoxicity assays showed insignificant effects on human immortalized keratinocyte cells, revealing the safety of the material for contact with the human skin.	[13]
			Cotton/polypyrrole-silver nanocomposites	Polymer–AgNPs nanocomposites modified cotton fabrics prepared by in situ chemical oxidative polymerization, displayed enhanced conductivity. AgNPs were also responsible for the increased antibacterial activity of the composite against *Staphylococcus aureus* and *Escherichia coli*.	[85]
	Silver and copper (Ag/Cu bimetallic NPs)	Inorganic particles with exceptional antimicrobial and antifungal properties.	Cotton/polyester composite	Cotton–polyester textiles were successfully impregnated during washing and ironing processes with five impregnation solutions containing Ag/Cu in the form of bimetallic NPs (alloy and core-shell) as well as ionic species. The antimicrobial activity of the fabrics was observed and did not become compromised after 20 washing cycles. Surfaces treated with solutions containing Ag^+^/Cu^2+^ and AgNPs/Cu^2+^ inhibited fungi growth significantly.	[86]
	Copper oxide (CuO)	Inorganic particles with antimicrobial properties. CuO has unique optical, catalytic and chemical properties at nanoscale.	Polycotton-based fabric	CuO-modified cotton fabrics revealed excellent resistance to microorganisms (bacteria and fungi) at different concentrations.	[87,88]
	Calcium carbonate (CaCO_3_)	Inorganic particles endowed with an ultra-fine solid structure and high economic value that play an important role in reinforcing and toughening materials and enhancing electrostatic attraction.	Kenaf fiber/polyester composite	Kenaf fiber–polyester composites produced via vacuum-assisted resin infusion process followed by CaCO_3_ NPs impregnation exhibited increased modulus of elasticity, modulus of rapture, tensile modulus and tensile strength, and a reduced swelling capacity and moisture absorption.	[89]
			Kenaf bast fibers-polyolefin matrices/polypropylene composite	CaCO_3_ was incorporated within the composite via the inorganic nanoparticle impregnation method. The tensile modulus and strength of the fibers increased significantly after NPs incorporation, as the compatibility of the modified kenaf fibers and polypropylene was significantly improved.	[90]
			Bamboo fiber/polypropylene composite	Impregnation of the bamboo fibers with CaCO_3_ increased the fiber density, filling the morphological voids and creases, and improving the interfacial compatibility of the composite. The modified composites exhibited improved tensile strength, modulus of elasticity, and elongation at break.	[91,92]
	Silver chloride (AgCl)	Like AgNPs, these inorganic particles are capable of great antimicrobial activity, by acting as leaching antibiotics.	Wool/polyester composite	Composites were prepared by pad-dry-cure method which generated a functional silica matrix that induced the in situ synthesis of AgCl NPs. Ag-modified surfaces were successful against bacteria and fungi at concentrations superior to 0.5 mM AgNO_3_.	[93]
	Silver zeolites (SZs)	Zeolites are crystalline aluminosilicates that exhibit adsorption properties and ion-exchange capabilities. By encapsulating silver, they allow an optimized release of the NPs and ensure antimicrobial activity without adverse effects.	Cotton/chitosan composites	Cotton fabrics were modified with a film of chitosan or by a conventional pad–dry–cure process in which chitosan–zeolite composites were immobilized onto the fabric surface. The altered fabrics displayed improved antibacterial properties against *Escherichia coli*, *Staphylococcus aureus*, *Candida albicans* and *Trichophyton rubrum*. Evidences of thermoregulating properties were also found.	[94]
	Zeolitic imidazolate framework-8 (ZIF-8)	Inorganic particles endowed with a large surface area, and strong hydrophobicity.	Cotton/ZIF-8-polydimethylsiloxane fabric	The modified cotton fabric showed superhydrophobic properties and excellent antibacterial action against *Escherichia coli* and *Staphylococcus aureus*. Fabrics retained their excellent antibacterial property and superhydrophobicity after 300 cycles of abrasion and 5 cycles of washing.	[95]
	Aluminum hydroxide (Al(OH)_3_)	Hydrophilic, inorganic particles, non-toxic and odorless that exhibit good dispersion and can generate very easily hydrogen bonds with cellulosic fibers.	Kenaf fibers/polyester composite	Kenaf fiber reinforced composites were produced via vacuum-assisted resin transfer molding process and impregnated with Al(OH)_3_ NPs. The NPs addition increased the composite modulus of elasticity, modulus of rupture, tensile modulus and tensile strength, while the water thickness of swelling was reduced.	[96]
	Titanium dioxide doped with iron and nitrogen atoms (TiO_2_)	Inorganic particles with photocatalytic activity, self-cleaning properties and base substrate-dependent superhydrophilicity/superhydrophobicity.	Cotton/reduced graphene oxide composite	Cotton fabrics treated with reduced graphene oxide were successfully decorated with two types of TiO_2_ NPs doped with 1% iron and nitrogen atoms and synthesized in different hydrothermal conditions. NPs-modified fabrics were found harmless for human skin cells and capable of inhibiting the growth of *Staphylococcus aureus* and *Enterococcus faecalis*.	[97]
	Iron oxide (magnetite, Fe_3_O_4_)	Inorganic particles with photocatalytic activity and antimicrobial properties.	Cotton/polyester composite	Sonosynthesis and sonofabrication of Fe_3_O_4_ NPs was accomplished on cotton/polyester composite fabrics, with appropriate saturation magnetization. Composites demonstrated a 95% antibacterial efficiency against *Staphylococcus aureus* and a 99% antifungal effect against *Candida albicans*, along with enhanced mechanical properties.	[98]
	Cerium oxide (CeO_2_)	Inorganic particles with outstanding catalytic, electronic and magnetic properties. They are also highly efficient in absorbing UV radiation and protecting against corrosion.	Chitosan/linen (from flax family) composite	Linen fabric was modified with chitosan followed by in situ synthesis of CeO_2_ NPs. The modified fabric displayed effective antibacterial activity against *Staphylococcus aureus* and *Escherichia coli* bacteria. They were also endowed with properties like wrinkle resistance, UV-protection and flame retardancy, which were maintained after 5 washing cycles.	[99]
	Platinum (Pt)	Inorganic particles very stable and effective for antimicrobial applications. PtNPs have high activity and selectivity for catalytic reaction, good recyclability, and can enhance the cleansing function of the skin surface.	Silk-based fabrics	PtNPs were synthesized in situ on silk-based fabrics through heat treatment. Color strength increased with the concentration of the Pt ions. The modified fabrics exhibited good washing fastness and excellent rubbing color fastness. They also demonstrated significant catalytic functions and a significant antibacterial effect against *Escherichia coli*.	[100]
	Bamboo	Biocompatible, organic particles endowed with superior mechanical properties, namely ultimate tensile, toughness and Young’s modulus.	Woven-nonwoven kenaf fiber/unsaturated polyester composite	Due to the high surface area of the bamboo NPs, incorporation allowed for a strong bond between kenaf and polyester to be generated with improved wettability and excellent mechanical and thermal properties.	[101]
**Enzymes**	Laccase	Laccases are multi-copper glycoproteins that catalyze the mono-electronic oxidation of phenols and aromatic or aliphatic amines to reactive radicals and reduce molecular oxygen to water in a redox reaction.	Lignocellulosic jute/polypropylene composite	Lignocellulosic jute fabrics were treated with laccase and then used as reinforcement materials to prepare polypropylene-based composites. Laccase-treated jute/polypropylene composites exhibited high breaking strength, storage modulus, and melting temperature. Data suggests a good interfacial adhesion between the jute and the polypropylene.	[102]
				Grafting of dodecyl gallate onto jute fibers via laccase was investigated as a reinforcement of polypropylene-based composites. The composite hydrophobicity and breaking strength increased after grafting, and the composite fracture section became neat and regular.	[103]
				Alkyl gallates with different aliphatic chain lengths, such as propyl gallate, octal gallate and dodecyl gallate, were enzymatically grafted onto jute by laccase and then incorporated onto polypropylene matrices. After modification, the tensile and dynamic mechanical properties of the composites improved, while water absorption and swelling decreased.	[104]
**Peptides**	RGD-peptide	Arginyl-glycyl-aspartic acid (RGD) is the most common and well documented peptide motif responsible for cell recruitment and attachment to the extracellular matrix.	Milkweed/polyethylene/polypropylene composite	A composite of milkweed, polyethylene and polypropylene was made by carding and further treated with atmospheric pressure plasma to functionalize the surface with carboxylic acid groups for RGD-peptide binding. Plasma treatment accelerated the degradation of milkweed. The composite was seen to promote MC3T3 osteoblast-like cells recruitment.	[11]
**Antimicrobial Peptides (AMPs)**	Cecropin-B/[Ala5]-Tritrp7	Cecropin-B is an antibacterial peptide found in the hemolymph of the pupae of H. cecropia. It is composed of 35–39 a.a. in length and assumes an amphipathic α-helice structure that facilitates microbial penetration. [Ala5]-Tritrp7 is a synthetic peptide that results from the replacement of the first Pro at position 5 in tritrpticin by Ala (Tritrp7). The substitution of Pro-5 to Ala in Tritrp7 leads to the formation of amphipathic α-helices, which stimulates an effective cell leaching and thus bacteria death.	Wool-based materials	AMPs immobilization was accomplished via exhaustion method. The functionalized AMPs reduced significantly the bacterial growth, with Cecropin-B resulting in 71.67% reduction against *Staphylococcus aureus* and 85.95% against *Klebsiella pneumoniae,* while [Ala5]-Tritrp7 promoted a 66.74% and 88.65% reduction, respectively.	[12]
**Plant Extracts**	Baicalin (5,6,7-trihydroxyflavone-7-O-glucuronid)	Major component of the root of *Scutellaria baicalensis* Georgi. It possesses multiple bioactivities including antibacterial, antioxidant, anticancer, anti-inflammatory, and antiviral activities.	Silk-based fabrics	Baicalin bonded with the silk fabric via electrostatic interactions between the ionized carboxyl groups in the extract and the positively charged amino groups in the fabric. The treated fabric exhibited excellent antioxidant activity, high antibacterial performance against *Escherichia coli* and *Staphylococcus aureus*, and very good UV-protection.	[105]
	Propolis	Propolis is a gum gathered by honeybees from various plants. It is not toxic to humans or mammals. Propolis has been reported as anticancer, antioxidant, anti-inflammatory, antibacterial, antifungal and antiviral.	Cotton-based fabrics	Cotton fabrics were treated with propolis at different concentrations using the pad–dry–cure technique. Surfaces were found highly antibacterial, water repellent and capable of protecting against UV-radiation.	[106]
	*Psidium guajava* Linn. leaf extract	*Psidium guajava* Linn., from the Myrtacae family, also known as guava, is characterized by its exceptional antidiabetic, anticough, antioxidant, antibacterial and antispasmotic properties.	Cotton-based fabrics	Microcapsules containing *Psidium guajava* Linn. leaf extract were prepared by in situ polymerization using urea and formaldehyde for encapsulation and applied by direct printing onto cotton fabrics. The extract modified fabrics showed antibacterial activity against *Staphylococcus aureus* but were not effective against *Escherichia coli* bacteria.	[107]
	Aloe Vera gel	Aloe Vera is a highly abundant, natural plant that has antimicrobial activity against various pathogens. External application of Aloe Vera gel penetrates the skin directly and produces a soothing, pain-relieving and anti-inflammatory effect on arthritic joints and tendonitis.	Cotton-based fabrics	Bleached cotton fabrics were immersed in the extracted solution for specific periods of time, padded, dried and cured. Modified fabrics became very effective against pathogens, namely *Bacillus subtillis*, *Pseudomonas aeruginosa*, *Bacillus pumalis* and *Escherichia coli*. The antimicrobial finishing did not affect the physical properties of the fabric.	[108]
	*Jatropha curcas* leaf extract	*Jatropha curca* is a plant indigenous of India composed of phenolic, terpenoids, flavonoids, alkaloids, glycosides, steroids, tannin, etc., which endows the extract with antibacterial properties (bactericide and bacteriostatic). It is also known for its anti-cancerous properties.	Cotton-based fabrics	An ecofriendly natural antibacterial finish was applied to cotton-based fabrics via dip coating. Modified fabrics were characterized as bactericides and bacteriostatic against *Staphylococcus aureus* bacteria.	[109]
	Curcumin	Bright yellow compound produced by *Curcuma longa* plants. It is endowed with many functions, including anti-inflammatory, anticancer, antiviral, antiarthritic and antioxidant properties.	Cotton and non-woven fabrics/diphenylalanine (FF) peptide nanotubes	Cotton and non-woven fabrics were decorated via sonochemical process with FF loaded with curcumin. A sustainable, controlled release of curcumin was attained using this functionalization process, which was modulated by the sonication time, conferring potential antimicrobial and anti-inflammatory properties to the fabric.	[110]
			Sisal fibers/poly(methyl methacrylate) composites	Composite microspheres loaded with curcumin and made of poly(methyl methacrylate) stabilized with cellulose nanocrystals prepared from sisal fibers were produced. Results showed curcumin loaded composites to display long-term photostability and good encapsulating ability.	[111]
	*Ocimum sanctum* leaf extract	*Ocimum sanctum* plant is found in India and has antibacterial, antioxidant, antibiotic, antiatherogenic, immunomodulatory, anti-inflammatory, analgesic, antiulcer, chemo-preventive and antipyretic properties. Besides it is very abundant and easily accessible, economically feasible, and possesses minimal side effects.	Cotton/polyester composite	The composite fabric was treated with the herbal extract at different concentrations, using glutaraldehyde as cross-linking agent and sodium hypophosphite as catalyst by the exhaustion method. Modified fabrics inhibited Gram-positive bacteria growth in more than 92%. Although, the treated fabrics showed enhanced crease recovery property, there was a marginal reduction in tensile properties.	[112]
**Essential Oils (EOs)**	Rosemary, lavender, clove and cinnamon	Bioactive oils endowed with antimicrobial properties.	Cotton/monochlorotriazinylβ-cyclodextin fabric	Cotton fabrics were modified with monochlorotriazinyl β-cyclodextrin, as an eco-friendly encapsulating/hosting compound, to create core-shaped hydrophobic cavities for individual loading of EOs. The modified fabrics revealed improved antibacterial activity and durability. The antibacterial activity of the treated knitted cotton fabrics was superior to that of woven fabrics.	[113]
	Citronella	Biopesticide with a non-toxic mode of action that works as a mosquito repellent due to its eco-friendly and biodegradable nature.	Wool/gelatin and gum Arabic biopolymers	Microencapsulation of citronella oil was done by complex coacervation onto wool fabrics. The multi-core structure of the microcapsules allowed the oil diffusion by a Fickian mechanism in the first release stage and by non-Fickian kinetics on the second stage. The textile structure influenced the release model due to the interaction between the fabric and water.	[114]
	Oregano	Oregano oil comes from the leaves and shoots of the oregano plant and is botanically known as *Origanum vulgare*. It is a natural antibiotic and antimicrobial agent with antioxidant, anti-inflammatory and anti-cancerous properties. It may also be involved in lowering cholesterol.	Sugarcane bagasse/starch foam composite	Sugarcane bagasse fiber-reinforced starch foam composites were prepared with different oregano essential oil contents. The addition of oregano oil increased the composite antimicrobial properties, particularly against Gram-positive bacteria, but decreases its water absorption capacity and hygroscopicity. The biodegradation rate and flexural strength of the composite slightly decreased with increasing oil content.	[115]
			Coconut fibers/poly(3-hydroxybutyrate-co-3-hydroxyvalerate) (PHBV) composite	Green composites were obtained by twin-screw extrusion followed by compression molding. Coconut fibers were impregnated with oregano essential oil by spray coating and then incorporated into PHBV. The green composites displayed enhanced physical performance and superior bacteriostatic effect against *Staphylococcus aureus* bacteria.	[116]
	Cinnamon	Cinnamon oil is derived from the bark or leaves of several trees, including the *Cinnamomum verum* tree and the *Cinnamomum cassia* tree. Possesses antibacterial, antifungal, antidiabetic and antioxidant properties.	Durian skin fiber/polylactic acid composite	Transparent composites were produced via solvent casting and further modified by the incorporation of cinnamon oil. Scanning calorimetry analysis showed that the oil-modified composites were less crystalline than the controls, suggesting their structure was less rigid and flexible. The oils decreased the water vapor permeability and improved the composite antimicrobial activity against Gram-positive and Gram-negative bacteria.	[15]

**Table 5:** Recent trends on biomolecule immobilization strategies onto natural fibers.

Natural Fiber	Cleaning	Pre-treatment	Immobilization Strategies	Functional Groups	Biomolecule	Main chemical Reactions	References
Flax	Non-ionic detergent 80 °C 30 min, DW 70 °C 30 min, 100 °C 10 min	-	Dip–pad–dry method to deposit pegylated silver NPs, drying 100 °C 20 min, water, drying 100 °C 6 min In situ NP synthesis by sol-gel method: immersion in Zn(CH_3_COO)_2_.2H_2_O 50 °C 1 h stirring, NaOH, drying 100 °C 6 h	-OH	Silver NPs and Zinc oxide NPs (inorganic NPs)	Metal–ligand binding with Ag^+^ and Zn^2+^ ions from NPs	[201]
Linen (flax family)	-	-	Dip–pad–dry–cure method: immersion in CA, NaPO_2_H_2_ and chitosan, padding, drying 100 °C 3 min, curing 140 °C 5 min *In situ* NP synthesis by sol-gel method: immersion in Ce(SO_4_)_2_ solution 45 min, NaOH 50 °C 30 min under ultrasound irradiation, cold water, drying	-OH	Chitosan (Polysaccharide) and Cerium oxide NPs inorganic NPs)	Esterification of linen with -COOH of CA; electrostatic interaction of CA with -NH_2_ of chitosan; Metal-ligand binding with Ce^3+^ ions	[99]
	-	-	Dip–pad–dry–cure method with chitosan, BTCA and NaPO_2_H_2_, dried 80 °C 4 min and cured 140 °C 4 min In situ NP synthesis by sol-gel method: immersion in AgNO_3_ 20 min, then in mordant TSCE 60 min under ultrasound irradiation, cold water, drying	-OH	Silver NPs (Inorganic NPs), Chitosan (Polysaccharide), *Tamarindus indica* L. seed coat extract (TSCE, plant extract)	Esterification with -COOH of BTCA; electrostatic interaction of BTCA with -NH_2_ of chitosan, and of -COOH, NH_2_ and -OH groups with silver nitrate; Metal–ligand binding between phenol groups of tannings of TSCE and Ag^+^ ions	[84]
Kenaf	-	-	Casting of a resin mixture (polyester resin with NP filler loadings and MEKP as catalyst) onto the fibers using hand layup process, cure cold press 24 h, polymerization 105 °C	-OH	Bamboo NPs (organic NPs)	Hydrogen bonding between NPs, fiber and matrix	[101]
Cotton		-	In situ NP synthesis by sol-gel process: immersion in Zn(NO_3_)_2_.6H_2_O and CH_3_C_3_H_3_N_2_H solutions in CH_3_OH 24h, DIW with ultrasound irradiation 10 min, drying 80 °C 2 h Immersion in THF solution with PDMS and curing agent stir 5 min, drying 80 °C 2 h	-OH	Metal–organic framework (zeolitic imidazolate framework-8, ZIF-8) (inorganic NPs)	Metal–ligand binding with Zn^2+^ ions	[95]
	-	Esterification through the dip–pad–cure–dry method: immersion in CMCS solution 15 min, pad-roll, cure 180 °C 5 min, DW, drying 100 °C 1 h. Same for Cys adsorption	In situ NP synthesis by sol-gel process: immersion in AgNO_3_ 10 min, drying 100 °C 1 h, immersion in NaBH_4_ 10 min, DW, drying 100 °C 1 h	-SH	Silver NPs (inorganic NPs)	Metal–ligand binding with Ag^+^ ions	[13]
	Ultrasound treatment in DIW, drying	Silanization: drying 55 °C 24 h, immersion in OTS and MTS in C_7_H_8_ sealed 10 min, drying	Immersion in silver NP dispersion for 10 min	-OH	Silver NPs (inorganic NPs)	Metal–ligand binding with Ag^+^ ions	[202]
	-	-	Ultrasound treatment: immersion into a hot dispersion of loaded FF peptide nanotubes in an ice bath, DW, freeze-drying	Unspecific	Curcumin (plant extract)	Physical adsorption after sonication process: based on the point melting of the substrate and carbonization of the fibers at the points of their contact with the silver nuclei due to the high rate and temperature of the nanotubes thrown to the solid surface by sonochemical microjets	[110]
	NaOH and C_58_H_118_O_24_ at 70 °C 20 min	Silanization: immersion in KH-580 solution 2 min, cure 120 °C 5 min	Thiol-maleimide click chemistry: immersion in CH_3_C(O)CH_2_CH_3_ with N-phenyl-male-imide and C_6_H_15_N 60 °C 30 min while stirring, drying 70 °C 10 min	-SH	N-phenyl-male-imide (organic compound)	Thiol-maleimide click chemistry	[203]
	NaOCl, DW, drying 60 °C 48 h	-	Immersion in amoxicillin solutions 10 min, drying 72 h fume hood Solvent casting technique: pouring of PLA solution in CHCl_3_ until submersion, solvent evaporation 72 h vacuum	-OH	Amoxicillin (antibiotic)	Hydrogen bonding and electrostatic interaction with cationic groups of amoxicillin like -NH_2_	[14]
	-	-	Deposition by extraction method		Poly(propylenimine) dendrimers from first and third generations modified with 1,8-naphthalimide units and their Zn(II) complexes (dendrimers)		[204]
	-	-	UV-photo-grafting method of alginate-Ca2+/PNIPAA hydrogel: PAAm, SA and other additives, UV 30 min, CaCl_2_ 24 h, DW	-OH	MB as model drug	Covalent bond with radical initiators that subtracted H atoms to cotton	[205]
	Acetone, DIW	Functionalization by immersion in dopamine solution at pH 8.5, DW, drying vacuum	In situ NP synthesis by sol-gel process: immersion in Zn(CH_3_COO)_2_ into CH_3_OH and NaOH 20 min, pad-rolled, dried in vacuum. Then, immersion into Zn(NO_3_)_2_.6H_2_O) and HMTA solutions 90 °C 5 h, DW, drying	Cathecol	Zinc oxide NPs (inorganic NPs)	Metal-ligand binding with Zn^2+^ ions	[206]
	Ultrasound treatment: C_12_H_25_NaO_3_S 30 min, ethanol 2 h, DIW 30 min 3 times	Dip–pad–dry–cure method: immersion in Cys30 min, pad, drying 3 min 80 °C, cure 180 °C 3 min, DW (3 times), drying 100 °C 1 h	In situ NP synthesis by sol-gel method: immersion in CuSO_4_ and CA 50 °C 30 min, NaBH_4_ 40 °C 1h, DW twice, drying 4 h	-SH	Copper NPs (inorganic NPs)	Metal–ligand binding between Cys on cotton and Cu^2+^ ions	[207]
	-	-	Pad–dry–cure process: immersion in chitosan-silver zeolite composites (previously obtained by ionic gelation method with TPP) at pH 5.5, drying 90 °C 3 min, crosslinked with CA 140 °C 2 min, water, drying	-OH	Silver zeolites	Esterification with -COOH of CA that also lead to chemical reaction with -NH_2_ of chitosan	[94]
	-	-	Pad–dry–cure technique: immersion in aqueous solution of ethanol extract liquid of propolis with glyoxal and Al2(SO4)3, padding, drying 80 °C 3 min, cure 140 °C 5 min, warm water 15 min, drying	-OH	Propolis (plant extract)	Covalent bond of -COH of glyoxal with -OH of propolis and fabric, hydrogen bonding, physical entrapment	[106]
	Turbo Break detergent (NaOH), Silex Emulsion detergent (fatty alcohol ethoxylates, NaOH), and Ozonit Performance detergent (CH_3_COOH, H_2_O_2_, CH_3_CO_3_H), Finale Liquid detergent (HCOOH)	-	Immersion in Ag_3_C_6_H_5_O_7_, C_4_H_6_O_4_Cu as precursors in water Immersion in mixed solution with C_4_H_6_O_4_Cu and Ag_3_C_6_H_5_O_7_, reduction with NaBH_4_, stabilizer PVP	-OH	Ag+/Cu2+ and Silver NPs/Cu2+ (inorganic ions, inorganic NPs)	Metal–ligand binding with Ag^+^/Cu^2+^ ions	[86]
Milkweed	Soxhlet extraction in acetone 24 h, vacuum-drying	Carding together with core-shell PE-coated PP fibers 80–120 °C Dielectric Barrier Discharge plasma treatment at atmospheric pressure	Immersion under stirring in EDC solution in MES buffer 30 min, MES buffer twice, RGD-TAMRA HEPES solution pH 7.4) 3 h, TWEEN-20 five times, DIW three times	-COOH	RGD (peptide)	Peptide covalent bond with NH_2_ with RGD peptide	[11]
Kapok	Filter, wash, drying	Functionalization by immersion in dopamine solution at pH 8 24 h	In situ NP synthesis by sol-gel method: immersion in AgNO_3_ UV irradiation under stirring 30 min, DW, drying vacuum	Catechol	Silver NPs (inorganic NPs)	Metal–ligand binding with Ag^+^ ions	[208]
Durian skin	Washing, chopping, grinding, drying and sieving	Solvent casting method: drying PLA and durian skin fiber, dissolution in ChCl3 while stirring, EPO, 24 h	Cinnamon oil addition to the previously formed composite	-OH	Cinnamon (essential oil)	Hydrogen and covalent bonding between the PLA/durian skin fiber and aldehydes in cinnamon oil	[15]
Bamboo	Ultrasound treatment: acetone, ethanol and DW, 15 min	Functionalization by immersion in dopamine solution at pH 8.5	In situ NP synthesis by sol-gel method: immersion in Ag_3_C_6_H_5_O_7_, microwave irradiation, rinse in DW, drying	Catechol	Silver NPs (inorganic NPs)	Metal–ligand binding with Ag^+^ ions	[209]
	Ultrasound treatment: water, detergent and Na_2_CO_3_, 1 h 60 °C	Air plasma treatment	Exhaustion bath with loaded microcapsules, Mikracat B crosslinking agent and Sapamine softener 1 h pH 7, padding, crosslinking 1 h 130 °C, drying	-COOH, -OH, -COH	Lavender oil (essential oil)	Covalent bonding between loaded microcapsules and fabric	[210]
	Water 70 °C 3 min, DW	-	In situ NP synthesis by sol-gel method: Immersion in HAuCl_4_, 15 min RT, 80 °C 60 min in oscillating water bath, DW, drying; or Immersion in AgNO_3_, 15 min RT, 80 °C 60 min in oscillating water bath, NaOH for pH 10, 80 °C 60 min, DW, drying	-OH	Gold and silver NPs (inorganic NPs)	Metal–ligand binding with Au^3+^/Ag^+^ ions	[211]
Silk	Water 50 °C, DW	-	In situ NP synthesis by sol-gel method: Immersion in H_2_PtCL_6_ at pH 5 10 min, 90 °C 60 min in shaking water bath, DW, drying. NaOH or CH_3_COOH to adjust pH to 6	-SH	Platinum NPs (inorganic NPs)	Metal–ligand binding between Cys on silk and Pt^+^ ions	[100]
	Warm water 5 min, DIW	-	In situ NP synthesis by sol-gel method: Immersion in HAuCl_4_ pH 3 20 min, 90 °C 60 min in shaking water bath, DIW, drying 70 °C; or Immersion in AgNO_3_ pH 10 20 min, 90 °C 60 min in shaking water bath, DIW, drying 70 °C	-SH	Gold and silver NPs (inorganic NPs)	Metal–ligand binding with Au^3+^/Ag^+^ ions	[212]
	-	-	Dip dyeing process: immersion dye solution pH 3 90 °C 60 min Mordant treatment with FeSO_4_, Fe_2_(SO_4_)_3_ and TiOSO_4_ 60 °C 30 min, tap water, drying	-SH	Tea stem extract (plant extract)	Electrostatic interaction with polyphenol groups of the extract	[213]
	3 times Na_2_CO_3_ boiling point 30 min, DW, drying		Exhaustion method: immersion in silver NP dispersion (previously reduced by SA) in shaking bath pH 4 40 °C 40 min, drying	- NH_2_	Silver NPs (inorganic NPs)	Electrostatic interaction with -COOH from SA	[214]
	--		In situ NP synthesis by sol-gel method: Immersion in AgNO_3_ 90 °C 3 °C /min from 30 °C, CfA, 90 °C 30 min with agitation, DIW, drying	-SH	Silver NPs (inorganic NPs)	Meta–ligand binding with Ag^+^ ions	[215]
	Three times Na_2_CO_3_ 98 °C 30 min, DW, drying	Layer-by-layer self-assembly: alternate immersion in PAH and PAA 3 °C 100 rpm 30 min followed by rinsing DW 1 min 3 times (outermost layer: PAH), drying 24 h	Immersion in heparin 4 °C 24h, PBS and DW under ultrasonic irradiation 10 min	-NH_2_	Heparin (polysaccharide)	Electrostatic interaction with sulfate groups of heparin	[216]
Wool	Non-ionic soap at 80 °C 20 min	-	Exhaustion method: in rota dyer, mordant treatment with TSCE 90 °C 60 min, squeeze, dyed with natural dye KFE 90 °C 60 min, cold water, dried	-CONH -OH	Kapok flower extract (plant extract) and Tamarind seed coat extract (TSCE, plant extract)	Bonding with phenol groups of tannings of TSCE and amide -CONH groups of wool; hydrogen bonding between mordanted wool and KFE	[217]
	-	-	Immersion in Cu(NO_3_)_2_ and C_6_H_3_(COOH)_3_ solution 85 °C, wash with DMF, drying	-SH -OH	Metal–organic framework-199 (HKUST-1, inorganic NPs)	Hydrogen bonding and Metal-ligand binding with Cu^2+^ ions	[218]
	Ultrasound treatment: acetone 3 h, drying 50 °C	-	Exhaustion method: immersion in LRM extract, warm water, cold rinse, drying 60 °C 15 min. Mordant treatment with FeSO_4_ and Fe_2_(SO_4_)_3_ 60 °C 30 min, rinse, drying	-OH	*Lycium ruthenicum* Murray extract (LRM, plant extract)	Hydrogen bonding and van der Waals forces with anthocyanin of the extract	[219]
	Soaking in water	Mordanting with KAl(SO_4_)_2_, FeSO_4_ and SnCl_2_ 91–93 °C 1 h under stirring, tap water	Immersion in natural dye solution 91–93 °C 1h manual agitation, non-ionic detergent Safewash, tap water, drying	-CONH	Pomegranate peel extract (plant extract)	Electrostatic interaction with phenolic compounds of dye	[220]
	Na_2_CO_3_ bath pH 8.5 60 °C 30 min and non-ionic detergent Nekanil 907, DW, drying	-	Exhaustion method: immersion in AMP solution 40 °C 1–3 h while stirring, 5-cycle washing with WOB detergent 40 °C 60 min, drying 37 °C 4 h	-COOH	Cecropin-B and [Ala5]-Tritrp7 (AMPs)	Electrostatic interaction with terminal -NH_2_ of peptides	[12]
	Non-ionic detergent Lotensol 60 °C 20 min	-	Exhaustion-dyeing process: immersion in dendrimer derivative dye 30 °C pH 5-5.5, 100 °C within 25 min + 60 min, non-ionic detergent 50 °C 20 min	-NH_2_	Poly(amidoamine) dendrimer (dendrimers)	Electrostatic interaction with terminal -COOH of dye molecules	[221]

**Abbreviations:** Ag_3_C_6_H_5_O_7_: silver citrate; AgNO_3_: silver nitrate; Al_2_(SO_4_)_3_: aluminum sulfate; AMP: antimicrobial peptide; BTCA: 1,2,3,4-Butanetetracarboxylic acid; CHCl_3_: chloroform; CH_3_C(O)CH_2_CH_3_: butanone; CH_3_CO_3_H: peracetic acid; C_4_H_6_O_4_Cu: copper (II) acetate; C_6_H_3_(COOH)_3_: trimesic acid; C_6_H_15_N: triethylamine; C_7_H_8_: toluene; C_12_H_25_NaO_3_S: sodium 1-dodecanesulfonate; C_58_H_118_O_24_: polyoxyethylene lauryl ether; CA: citric acid; Ce(SO_4_)_2_; CfA: caffeic acid; CH_3_C_3_H_3_N_2_H: 2-methylimidazole; CH_3_OH: methanol; CMCS: carboxymethyl-chitosan; Cu(NO_3_)_2_: copper nitrate; CuSO_4_: copper sulfate; Cys: L-cysteine; DIW: dionized water; DMF: dimethylformamide; DW: distilled water; EDC: 1-ethyl-3-(3-dimethylaminopropyl)carbodiimide hydrochloride; EPO: epoxidized palm oil; FF: diphenylalanine; Fe_2_(SO_4_)_2_: ferric sulfate; FeSO_4_: ferrous sulfate; HAuCl_4_: tetrachloroauric acid; HCOOH: formic acid; H_2_PtCl_6_: chloroplatinic acid; HMTA: hexamethylenetetramine; ITX: 2-isopropylthioxanthone; KAl(SO_4_)_2_: potash alum; KH-580: silane coupling agent; MB: methylene blue; MEKP: methyl ethyl ketone peroxide; MES: 2-(N-Morpholino)ethanesulfonic acid; NIPAAm: N-isopropylacrylamide; NP: nanoparticle; Na_2_CO_3_: sodium carbonate; NaBH_4_: sodium borohydride; NaPO_2_H_2_: sodium hydrophosphite; NaOCl: sodium hypochlorite; NaOH: sodium hydroxide; OTS and MTS: long and short silanes; PDMS: polydimethylsiloxane; PE: polyethylene; PLA: polylactic acid; PP: polypropylene; PVP: polyvinylpyrrolidone; RGD: arginylglycylaspartic acid; SA: sodium alginic acid; SnCl_2_: stannous chloride; TAMRA: carboxylic acid of tetramethylrhodamine THF: tetrahydrofuran; TiOSO_4_: titanium sulfate; TPP: sodium tripolyphosphate; Zn(CH_3_COO)_2_.2H_2_O: zinc acetate dihydrate; Zn(NO_3_)_2_.6H_2_O: zinc nitrate hexahydrate.
